# Inequitable Housing Practices and Youth Internalizing Symptoms: Mediation Via Perceptions of Neighborhood Cohesion

**DOI:** 10.17645/up.v7i4.5410

**Published:** 2022-10-27

**Authors:** Richard C. Sadler, Julia W. Felton, Jill A. Rabinowitz, Terrinieka W. Powell, Amanda Latimore, Darius Tandon

**Affiliations:** 1 Division of Public Health, Michigan State University, USA; 2 Department of Family Medicine, Michigan State University, USA; 3 Center for Health Policy & Health Services Research, Henry Ford Health System, USA; 4 Department of Mental Health, Johns Hopkins Bloomberg School of Public Health, USA; 5 Department of Population, Family, and Reproductive Health, Johns Hopkins Bloomberg School of Public Health, USA; 6 Johns Hopkins Bloomberg School of Public Health, USA; 7 Center for Addiction Research and Effective Solutions, USA; 8 Center for Community Health, Northwestern University Feinberg School of Medicine, USA

**Keywords:** anxiety, Baltimore, blockbusting, depression, gentrification, internalizing symptoms, neighborhood social cohesion, redlining

## Abstract

Disordered urban environments negatively impact mental health symptoms and disorders. While many aspects of the built environment have been studied, one influence may come from inequitable, discriminatory housing practices such as redlining, blockbusting, and gentrification. The patterns of disinvestment and reinvestment that follow may be an underlying mechanism predicting poor mental health. In this study, we examine pathways between such practices and internalizing symptoms (i.e., anxiety and depression) among a sample of African American youth in Baltimore, Maryland, considering moderation and mediation pathways including neighborhood social cohesion and sex. In our direct models, the inequitable housing practices were not significant predictors of social cohesion. In our sex moderation model, however, we find negative influences on social cohesion: for girls from gentrification, and for boys from blockbusting. Our moderated mediation model shows that girls in gentrifying neighborhoods who experience lower social cohesion have higher levels of internalizing symptoms. Likewise for boys, living in a formerly blockbusted neighborhood generates poorer social cohesion, which in turn drives higher rates of internalizing symptoms. A key implication of this work is that, in addition to standard measures of the contemporary built environment, considering other invisible patterns related to discriminatory and inequitable housing practices is important in understanding the types of neighborhoods where anxiety and depression are more prevalent. And while some recent work has discussed the importance of considering phenomena like redlining in considering long-term trajectories of neighborhoods, other patterns such as blockbusting and gentrification may be equally important.

## 1. Background

The links between urban development and mental health are well-established. Internalizing symptoms (including anxiety and depression) are relatively common across adolescence (Merikangas et al., 2010), but minority youth living in cities may be at heightened risk due to the higher crime rates and disadvantages (e.g., lower parental wealth, home ownership, residential stability) that often characterize their communities (Dupéré et al., 2012; Formoso et al., 2010). This may in part be because of the added stress of living in distressed communities, and how stress contributes to anxiety and depression (Wallace, 2012). Such symptoms have been predictive ofearly-onset substance use/misuse (King et al., 2004), suicidality (Nock et al., 2006), and other risky behaviors (Wickrama & Wickrama, 2010). Consistent with life course and social determinants of health theories, discriminatory and racist housing practices may affect youth’s outcomes across development and have upstream effects on mental health. Historical and contemporary discriminatory practices—such as redlining, blockbusting, and gentrification—have shaped and continue to shape the social and material resources available to minority youth living in disinvested urban communities. In turn, these disparities in access to resources may influence the way children experience and cope with stressors.

While redlining was outlawed in 1968, the decades-long practice of excluding minorities from access to mortgages created huge gaps in accrued wealth. The ensuing practice of blockbusting and white flight—generated by the mortgage and banking industry in the 1970s and 1980s to spark panic selling and “flip” previously all-white neighborhoods (Gotham, 2000)—led to a massive shift of resources in communities. While now also illegal in practice, the legacy of blockbusting contributes to rates of predatory lending and continued racial steering today (Kahrl, 2017). More recently, other inequitable forms of redevelopment—whereby investments are made in communities that often do not benefit existing minority residents—continue the pattern of uneven urban development (Gotham, 2002) that shapes place-based health disparities.

Disinvested urban environments trend with higher rates of anxiety and depression among youth (Cooley-Strickland et al., 2011; Rathus et al., 1995; Von Nebbitt et al., 2008), though elements of connectedness like social cohesion may buffer against the negative outcomes felt in such neighborhoods. Thus, being in a community where cohesion is hard to form—or where the built environment creates daily stressful experiences—may increase the levels of internalizing symptoms among youth in neighborhoods where redlining or blockbusting once took place, or where gentrification is currently occurring (and these may operate differently across neighborhood types). Given the negative behavioral health outcomes associated with internalizing symptoms, further inquiry regarding the potential impact of inequitable and discriminatory housing practices and inequities within the built environment is necessary to inform interventions aimed at reducing these symptoms and related sequelae.

In the current study, we examined: (a) whether inequitable housing practices—specifically redlining, blockbusting, and gentrification—were associated with internalizing symptoms, (b) whether these practices predict levels of neighborhood cohesion for boys and girls, and (c) whether neighborhood social cohesion mediated the relation between these neighborhood practices and internalizing symptoms. We examine all three practices in this article because the associations between the processes and outcomes are likely distinct, and merit consideration.

Such practices are essential to consider and correct because the decades-long processes of structural racism in housing make us more vulnerable to the impacts of climate and urban change (Saign, 2021; Toolis, 2021). American cities are weaker by way of these practices, which artificially depressed densities and property values, making the provision of basic city services more difficult (Kaplan & Sommers, 2009; Lee, 1996; Ross & Leigh, 2000; Rugh & Massey, 2010). The ways we have harmed our cities and the people in them undoubtedly negatively impact the progress we should be making toward achieving World Health Organization-recommended sustainable development goals for healthy cities. By quantifying and understanding how such structural racism disadvantages urban residents, we will be better equipped to build healthier cities in the future that account for past wrongs by maintaining a focus on equity and justice.

### 1.1. Inequitable Housing Practices in the US

#### 1.1.1. Redlining and Blockbusting

Redlining and blockbusting are two inequitable housing practices that have upheld racial and economic residential segregation and are responsible for vast differences in the quality of the built environment. Prior to 1968, no federal law ensured fair housing for all races (Kanter, 1993). Starting in the 1930s, the Home Owners’ Loan Corporation formalized an exclusionary practice known as redlining. This in effect provided a basis on which many agencies withheld loans to people living in neighborhoods considered to be too high a financial risk (Hillier, 2003). The practice of categorizing neighborhoods according to their suitability for receiving mortgages was, in practice, racist. While redlined areas included a mix of white, black, and other minority neighborhoods, almost every majority African American neighborhood was redlined (Michney, 2021; Sadler et al., 2020). Though research examining the impact of living in areas with a history of discriminatory housing practices on mental health is limited, a recent study showed that adults living in areas with a prior history of redlining were more likely to report poorer health outcomes including cancer, diabetes, obesity, stroke, and poorer mental health (Nardone et al., 2020).

Following redlining’s prohibition, blockbusting was used to maintain segregation. Real estate agents and the mortgage industry colluded to create panic selling in previously all-white neighborhoods, convincing white residents to sell low and re-selling these homes at a premium to African American and other minority families (Highsmith, 2012). Blockbusting reproduced the segregated neighborhoods common to the redlining era, leading to massive disinvestment in previously middle-class neighborhoods (Sadler & Lafreniere, 2017).

In addition to the direct financial implications of housing discrimination (Priester et al., 2017), it also causes “pain, hurt, humiliation, and insult” (Heinrich, 1992, p. 52) and negative health outcomes among residents (Yang et al., 2016). Because housing discrimination can take new forms in spite of policy change (e.g., the shift from redlining to blockbusting to more contemporary patterns like gentrification), it has been referred to as a moving target (Massey, 2005). Continued inquiry into its various forms—and their impact on mental health—is therefore of importance.

#### 1.1.2. Gentrification

Gentrification commonly entails an in-migration of middle-income residents, while spurring displacement of lower-income residents (Mujahid et al., 2019). It often includes new investments in communities (many of which are poor or formerly segregated) which bring increased institutional resources, improved mechanisms for informal social control, and improved academic and employment outcomes (Formoso et al., 2010). Gentrification is not as explicitly racist as past practices, because it is a function of market-driven urban policy that favors market-rate housing, relegates social problems, and reshapes social-cultural patterns in cities (Fraser et al., 2013). Even so, the inequality and displacement it creates make it similar to segregation in some ways (Wyly & Hammel, 2004).

A number of studies have linked gentrification with myriad health outcomes, although findings are mixed (Schnake-Mahl et al., 2020). Gentrification can negatively impact the well-being of existing residents by (a) displacing residents and businesses alike resulting in social network disruptions, (b) exacerbating income inequality between existing and incoming residents, and (c) adding stress to existing residents both by way of social marginalization for those remaining in place and displacement for those unable to afford to stay (Elliott-Cooper et al., 2020; Formoso et al., 2010; Wilder et al., 2017). Households that are displaced may also experience financial hardships of relocating and lose access to institutions (e.g., school) or other resources (e.g., job; Schnake-Mahl et al., 2020).

However, there is also evidence that gentrification may contribute to better health outcomes. For example, increased empowerment for community improvement and cross-cultural exchange may bring new opportunities (Wilder et al., 2017). Moreover, as noted by Schnake-Mahl et al. (2020), gentrification may result in greater economic opportunities, increased safety, and increased access to resources (e.g., health care services, green spaces). Indeed, there is evidence that economically or physically vulnerable adults in gentrifying neighborhoods have reported experiencing better health than those in unchanging low-income neighborhoods (Smith et al., 2017). Given these mixed findings, further investigation of gentrification’s effects is warranted.

### 1.2. Inequitable Housing Practices and Internalizing Symptoms

Inequitable housing practices uphold racial and economic residential segregation and influence unequal access to resources and treatment (White & Lawrence, 2018) that create disparities in multiple health outcomes (Acevedo-Garcia et al., 2003; Mendez et al., 2014; Shaw et al., 2010). Beyond negative interpersonal outcomes, the effects of discrimination shape the environments where we live and the opportunities people have for employment, education, and social interaction (Williams et al., 2019).

While segregation may have a protective effect on mental distress via living among one’s own group (Nobles et al., 2017), it typically entails unequal access to resources (Do et al., 2017; Kwate, 2017). The incurred time burden involved in accessing resources precludes residents from investing their time in other activities (work, social/family life, education), and can not only reinforce cycles of poverty but also compromise mental health and potentially make individuals feel helpless and defeated (Beidas et al., 2012; Hurd et al., 2013).

People in segregated or resource-scarce neighborhoods may also internalize these environments as a personal deficit instead of seeing the structural racism that caused it, which may contribute to feelings of depression and anxiety. Conversely, racist societal assumptions contribute to the racial empathy gap and implicit bias that is negatively experienced among minority populations. Ethnic density can lessen depressive symptoms to a point but is shown to contribute to higher levels in highly segregated neighborhoods (Bécares et al., 2014). Additional evidence suggests that segregation and residential instability both negatively contribute to mental health among children (Alegría et al., 2015; Jones et al., 2019). The instability caused by gentrification may have similar negative impacts as well. Taken together, the limited research examining the impact of historical neighborhood practices on internalizing symptoms is unclear and warrants further investigation to inform the development of interventions aimed at attenuating youth internalizing symptoms.

Where new investments do take place (often in areas considered to be gentrifying), the resulting housing inequalities may confer risk for internalizing symptoms among existing youth. For example, research suggests that more affluent older adults reported higher levels of anxiety and depressive symptoms relative to adults living in more economically depressed areas which may be due to concerns over increases in the cost of living, and anxiety regarding housing displacement and closure of businesses (Smith et al., 2017).

### 1.3. Neighborhood Social Cohesion and Internalizing Symptoms

Neighborhood social cohesion is defined by one’s sense of community, neighborly trust, and the positive social interactions that occur therein (Buckner, 1988; Robinette et al., 2018). While historical processes play a role in shaping internalizing symptoms, perceived contemporary neighborhood cohesion and connectedness may greatly influence these relations. Individuals in low-income neighborhoods who also perceive their neighborhoods as less cohesive are more likely to experience anxiety and depression (Kingsbury et al., 2015; Rabinowitz et al., 2016). In contrast, cohesion moderates the relation between neighborhood disadvantage and depressive symptoms (Dawson et al., 2019) such that higher levels of cohesion and collective efficacy contribute to a slower onset of internalizing symptoms in disadvantaged communities (Browning et al., 2013; Glasheen et al., 2014; Pearson et al., 2019).

Indeed, research suggests that social connectedness may buffer against the negative impacts of gentrification, particularly among vulnerable populations (Fong et al., 2019), although some work suggests that gentrification can be beneficial for promoting collective efficacy (Steinmetz-Wood et al., 2017). But because new development often rapidly prices out the most vulnerable populations, polarizes the social structure, and undermines social cohesion, these benefits are not always seen, and we, therefore, cannot assume only positive effects from gentrification (Butler, 2003; Cole et al., 2017; Uitermark et al., 2007). Conversely, the social structures of close-knit communities may also predispose a greater likelihood of internalizing symptoms among children via excessive parental monitoring (Kingsbury et al., 2015). Yet other forms of connectedness—such as inter-generational closure, where social networks between youth extend to their parents—may also be protective against developing internalizing symptoms (Formoso et al., 2010). One other potential mechanism related to neighborhood change is that moving out of violent, low collective efficacy neighborhoods can have a beneficial impact on adolescents’ self-efficacy (Dupéré et al., 2012), which has been predictive of decreases in internalizing symptoms (Singh & Bussey, 2011). Thus, even when residents are displaced, perceiving one’s neighborhood as cohesive may be associated with attenuated internalizing symptoms.

### 1.4. Sex Differences, Internalizing Symptoms, and Neighborhood Variables

While it is possible that historical neighborhood practices and perceived neighborhood cohesion may impact internalizing symptoms, there is reason to believe that sex differences may impact these relations. For example, living in a low-income neighborhood has been shown to predict social anxiety for girls, but not boys (Vine et al., 2012). Additionally, neighborhood disorder positively predicted internalizing symptoms for girls (Browning et al., 2013), including depression, anxiety, and autonomic arousal (Hill et al., 2005). Conversely, for boys, no associations were found between disorder and collective efficacy in internalizing symptoms (Browning et al., 2013).

In addition, girls may be impacted more by their environments than boys (Milam et al., 2012). For example, research suggests that boys may experience lower levels of anxiety and depression upon moving out of their neighborhoods relative to girls (Leventhal & Brooks-Gunn, 2003). Moreover, lower levels of neighborhood crime have been associated with lower internalizing symptoms among boys, but not girls (Rabinowitz et al., 2016). Although the mechanisms through which neighborhood practices may affect boys and girls differently are unclear, it has been hypothesized that the effects of neighborhood social cohesion may be more pronounced in girls relative to boys as girls tend to be more affiliative than boys (Frydenberg & Lewis, 1991), and are more likely to seek out social support from others when exposed to stressors (Piko, 2001).

### 1.5. Current Study

To address the existing gaps in the literature, the current study examined pathways between inequitable housing practices and internalizing symptoms and the differential impact of these effects on boys and girls. We hypothesized that (a) there would be a direct and positive effect of inequitable housing practices on internalizing symptoms, (b) housing practices would also predict neighborhood cohesion and that these relations would be further moderated by sex, and, finally, (c) neighborhood cohesion would mediate the relation between housing practices and internalizing symptoms in both boys and girls.

## 2. Methods

Participants were predominantly African American youth from Baltimore, Maryland, originally recruited for the Youth Opportunity (YO) program. The YO program’s goals are to increase access to educational, occupational, and training opportunities for adolescents and young adults (Sonenstein et al., 2011). The YO program was implemented in two neighborhoods (in East and West Baltimore), but participants came from 49 of Baltimore’s 55 community statistical areas (also referred to here as neighborhoods). Inclusion criteria required youth to be between 16 and 23 years old and not be in foster care. Informed consent and assent were obtained from adult and youth participants, respectively. The study was approved by the Johns Hopkins University School of Medicine Institutional Review Board. A more detailed description of the YO program and study design is detailed elsewhere (Sonenstein et al., 2011; Tandon et al., 2015).

Data were collected at three time points: baseline (when the study began in 2008), six months post-baseline, and one to two years post-baseline. Baseline data for the current study included 782 youth (51.0% female; 93.7% African American; *M*_age_ = 18.76, *SD* = 1.71). Given the very small percentage of the sample that was not African American, only African Americans were included in the analyses (*N* = 733; 51.0% female; *M*_age_ = 18.75, *SD* = 1.71, range: 16–23). Approximately 60% of the sample participated in one of the YO programs and about 10% of the sample reported being employed (see Table 1 for additional sample information and descriptive statistics).

### 2.1. Measures

#### 2.1.1. Neighborhood Social Cohesion

We assessed neighborhood social cohesion using a three-item scale developed by Kerrigan et al. (2006). One item, for example, is “people in my neighborhood are willing to help each other.” Items were rated on a four-point Likert scale (1 = *strongly agree* to 4 = *strongly disagree*), reverse coded, and summed with higher scores reflecting higher levels of social cohesion. In the current sample, the measure demonstrated excellent internal reliability (coefficient alpha = 0.75).

#### 2.1.2. Inequitable Housing Practices

**Figure 1.**
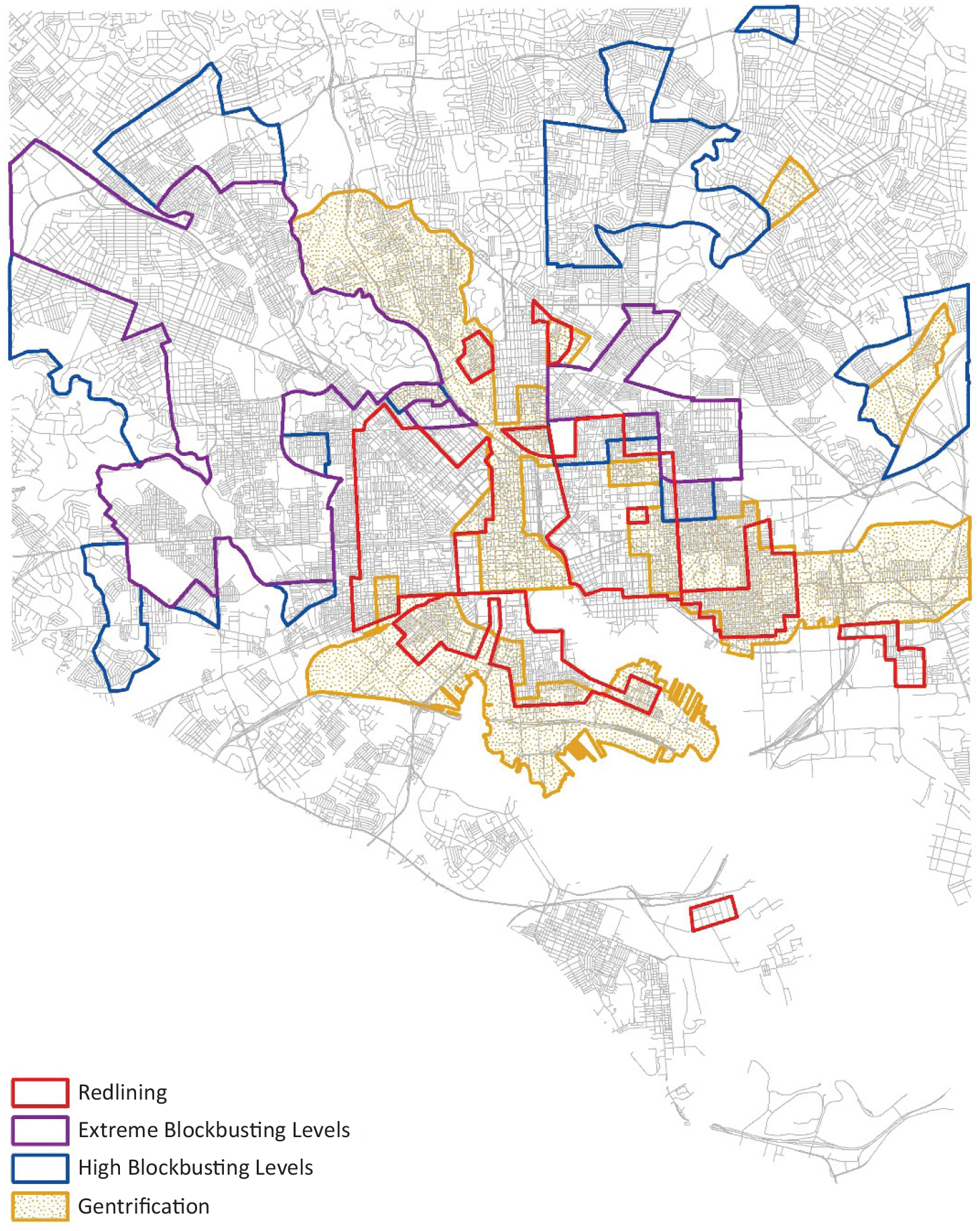
Map of Baltimore illustrating locations of inequitable housing practices.

For each participant, we joined variables denoting whether they lived in an area that had been redlined, blockbusted, or gentrified (a summary map is included in Figure 1). We used geographic information systems (GIS) software to join each participant to their neighborhood (defined as their community statistical area) and appended characteristics from that neighborhood to the participant.

Gentrification was measured according to the metric created by the National Community Reinvestment Coalition, which incorporates socioeconomic and demographic changes from the 2000 to 2010 censuses (Richardson et al., 2019). Socioeconomic changes are measured as increases above the 60th percentile in median home value and college-educated population. Demographic changes are measured as a 5% or greater decline in the predominant racial/ethnic group, or a decline in the percentage of the population of more than two standard deviations from the national mean. This metric is of interest here because Baltimore has one of the highest rates of gentrification in the US (Richardson et al., 2019). Neighborhoods were classified by the percentage of land area that fell within a gentrified census unit. Although our sample is not longitudinal, we assume that most youths in gentrifying neighborhoods are not in the incoming, higher-income group, based on the nature of the program from which our sample was drawn.

Redlining was measured according to the original metric created by the Home Owners’ Loan Corporation in the 1930s. We digitized the redlining maps and out-lined all areas that fell within a “red” zone. We then overlapped the redlining variable with the neighborhoods, and, like gentrification, neighborhoods were classified by the percentage of land area that fell within a formerly redlined neighborhood. These metrics reflect GIS-based practices for redlining in past work (Hillier, 2003; McClure et al., 2019; Sadler & Lafreniere, 2017).

Unlike gentrification and redlining, blockbusting has not commonly been considered as a potential determinant of contemporary health disparities or negative mental health outcomes. In fact, prior to Sadler and Lafreniere (2017), no study had operationalized a definition of blockbusting for GIS-based inquiries. Thus, here we replicate their procedure for identifying potentially blockbusted neighborhoods. We calculated the percentage of change in the white population in the census periods between 1950 and 1980. Neighborhoods where a majority of the white population moved within one decade (>50%) are considered to have been blockbusted. These values were then overlapped with neighborhoods as the gentrification and redlining variables had been, and neighborhoods were assigned the percentage of land area that was blockbusted.

#### 2.1.3. Internalizing Symptoms

Internalizing symptoms were assessed by creating a composite of measures of anxiety and depressive symptoms. Anxiety symptoms were evaluated using the BAI (Beck & Steer, 1990). The BAI is a 21-item measure that assesses physiological, behavioral, and cognitive indicators of anxiety. One item, for example, is “during the past month, how much have you been bothered by a fear of losing control?” Items were rated on a three-point Likert scale (1 = *mildly but it didn’t bother me much* to 3 = *severely—it bothered me a lot*) and summed to create a composite score (*α* = 0.90). The measure demonstrated excellent internal reliability in the current sample (coefficient alpha = 0.89).

Depression symptoms were assessed using the CES-D (Radloff, 1977). The CES-D assesses four main constructs including depressed affect, anhedonia, somatic activity, and interpersonal difficulties. Participants were asked to rate how they felt or behaved in the past week, such as whether they felt fearful or that their life had been a failure. Items were rated on a four-point Likert scale (0 = *rarely or none of the time* to 4 = *most or all of the time*) and summed to create a composite score (*α* = 0.86). In the current sample, the measure demonstrated internal reliability (coefficient alpha = 0.79). The anxiety and depression composites were *z*-scored (*M* = 0, *SD* = 1) and summed to create an internalizing symptom composite (coefficient alpha = 0.81).

### 2.2. Statistical Analyses

**Figure 2.**
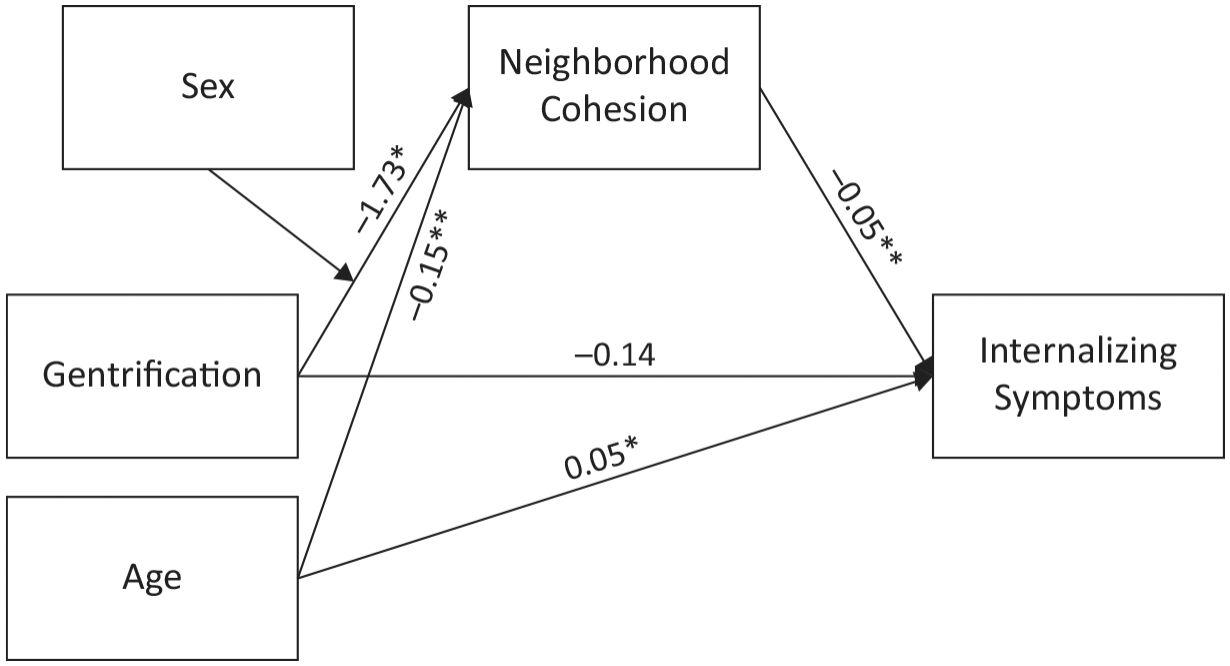
Model depicting mediation pathways linking gentrification, neighborhood cohesion, and internalizing symptoms. Note: *p < 0.05, **p < 0.01.

Patterns of missing data and univariate normality were examined for all variables. Means and standard deviations between key study variables were also evaluated. Inequitable housing practices were examined in separate models to reflect the distinct time periods and investment patterns of each practice. Each of the hypotheses regarding the effects of inequitable housing practices was tested in a series of main effect and mediation models. First, the direct effects of sex, age, and each of the three inequitable housing practice predictors (i.e., redlining, blockbusting, and gentrification) on internalizing symptoms were evaluated. Second, the main effects of housing practices on neighborhood cohesion were examined. Next, we examined whether these relations were moderated by sex. Finally, we evaluated a moderated mediation model in which we examined whether the indirect pathway from inequitable housing practices to internalizing symptoms via neighborhood cohesion differed for boys and girls (see Figure 2).

All analyses were run in SPSS Version 24 using the PROCESS macro. Non-parametric bootstrapping procedures (repeated, random sampling with replacement of indirect effect estimates) were utilized to evaluate the significance of the indirect effects as well as examine an index of moderated mediation. Unlike hypothesis testing based on parametric statistics, bootstrapping procedures do not assume that the indirect effect (the product of the effect of the independent variable to the mediator and the effect of the mediator on the outcome) is normally distributed (Preacher & Hayes, 2008). Indirect effects estimates with 95% bootstrapped confidence intervals that do not include zero indicate a statistically significant mediation effect.

## 3. Results

Very low rates of missing data were found for each variable (0–3.7%). All dependent variables were found to be within acceptable ranges for skew and kurtosis (≤3.0). The correlations between blockbusting and both redlining (*r* = −0.39) and gentrification (*r* = −0.31) were moderate and negative, while the relation between redlining and gentrification was moderate and positive (*r* = 0.35). Results for the primary analyses are presented below.

### 3.1. Direct Effects of Inequitable Housing Practices on Internalizing Symptoms

Our first set of models evaluated the effect of redlining, gentrification, and blockbusting as predictors of internalizing symptoms (controlling for participant age and sex) in three separate models. Across all models, only sex and age were significant predictors of internalizing symptoms, which indicated that girls and older youth experienced greater levels of symptomatology.

### 3.2. Main Effects of Inequitable Housing Practices on Neighborhood Cohesion

In our second set of models, we examined inequitable housing practices as predictors of neighborhood cohesion in three separate models. In each of the models, only younger age was consistently linked to greater perceived neighborhood cohesion. Sex was also associated with neighborhood cohesion, indicating that boys reported higher levels of perceived neighborhood cohesion. None of the inequitable housing practices was a significant predictor of cohesion.

### 3.3. Moderation Models

**Figure 3.**
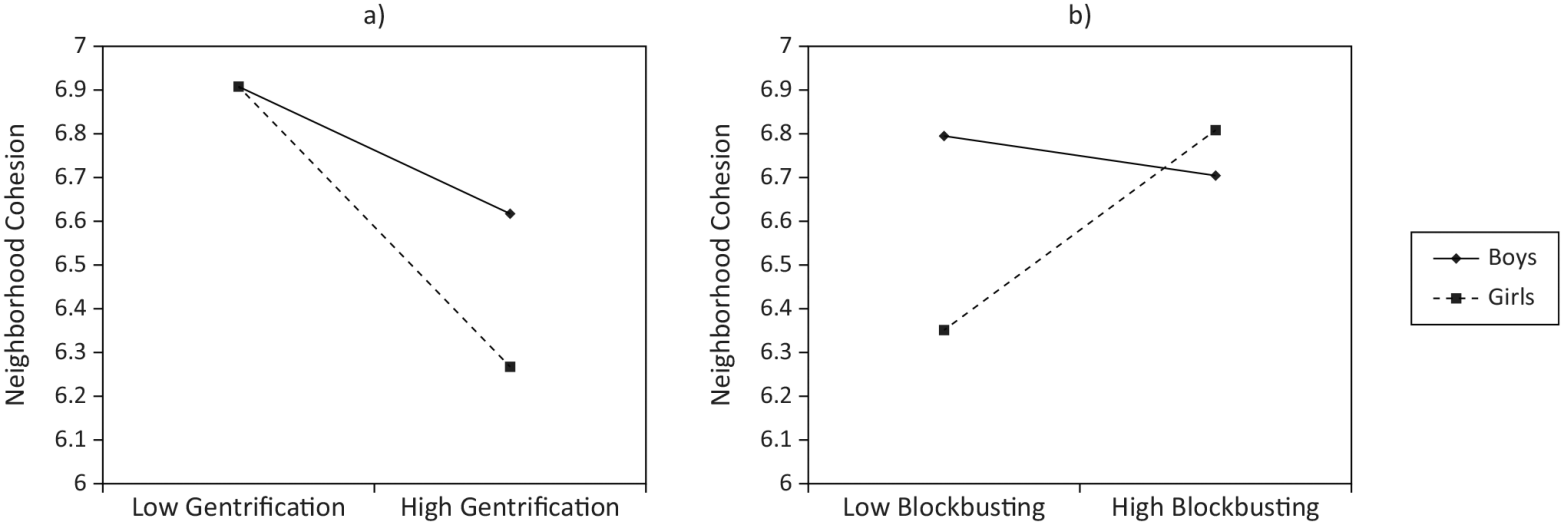
Plots of two-way interactions between sex and (a) gentrification and (b) blockbusting.

Next, we examined whether sex moderated the pathway between inequitable housing practices and neighborhood cohesion in three separate models (controlling for participant age). The first model found a marginally significant interaction between gentrification and sex (*β* = −0.233, *p* = 0.050), such that there was a negative effect of gentrification on neighborhood cohesion for girls only (see Table 2). In other words, girls in gentrified neighborhoods reported lower perceived neighborhood cohesion (see Figure 3). We also found a significant interaction between sex and blockbusting (*β* = 0.400, *p* = 0.002); however, this effect was in the opposite direction. Results suggest a significant, negative effect of blockbusting on neighborhood cohesion for boys only, indicating that boys who lived in areas with higher rates of blockbusting reported less neighborhood cohesion (see Figure 3). Finally, there was not a significant interaction between sex and redlining predicting neighbourhood cohesion.

### 3.4. Moderated Mediation Models

Finally, we examined a series of moderated mediation models. To examine the impact of gentrification on internalizing symptoms via neighborhood cohesion, we first conducted a mediation model controlling for participant age and sex. We did not find a significant direct effect of gentrification on internalizing symptoms, nor a significant indirect effect through neighborhood cohesion. We then added sex as a moderator of the pathway from gentrification to neighborhood cohesion and conducted a moderated mediation model predicting internalizing (controlling for participant age). Results support a moderated mediation model, indicating a significant indirect effect of gentrification on internalizing symptoms for girls only (*IE* = 0.06, 95% bootstrapped CI = 0.01 to 0.14). Moreover, the difference between the indirect effects for boys and girls was statistically significant (index of moderated mediation = 0.08, 95% bootstrapped CI = 0.01 to 0.18). These findings indicate girls exposed to higher rates of gentrification experienced lower neighborhood cohesion which, in turn, predicted elevated levels of internalizing symptoms (see Figure 2).

Our next set of models evaluated a mediation model in which exposure to blockbusting predicted neighborhood cohesion which, in turn, predicted internalizing symptoms (controlling for participant age and sex). Findings suggest that the direct effect of blockbusting on internalizing symptoms was not significant, nor was the indirect effect via neighborhood cohesion. We then added sex as a moderator of the pathway from blockbusting to neighborhood cohesion within the larger mediation model (continuing to control for participant age). Results indicate that a significant interaction effect between sex and blockbusting predicting neighborhood cohesion in the same pattern as reported above. Findings suggest a significant moderated mediation effect, indicating that living in a historically blockbusted area was associated with lower neighborhood cohesion for boys only and that this, in turn, predicted higher rates of internalizing symptoms (*IE* = 0.04, 95% bootstrapped CI = 0.01 to 0.08). The index of moderated mediation was also significant (–0.06, 95% bootstrapped CI = −0.12 to −0.02) suggesting these differences were statistically significantly different between boys and girls.

We then examined the same series of models using redlining as a predictor. Looking first at the relation between redlining and internalizing symptoms via neighborhood cohesion (controlling for participant age and sex), we found no direct or indirect effect of redlining on internalizing symptoms. We then considered a moderated mediation model (controlling for participant age). We found that sex did not moderate the pathway from redlining to neighborhood cohesion and that there was not a significant moderated mediation effect. All moderated mediation models were also run controlling for each of the other housing practices. An identical pattern of results emerged.

## 4. Discussion

Our first major finding is that girls living in gentrifying neighborhoods reported lower perceived neighborhood cohesion, which in turn predicted elevated levels of internalizing symptoms. The fact that girls in gentrifying neighborhoods experienced greater levels of internalizing symptoms suggests that neighborhoods may fail to incorporate some existing residents into the new and changing social life of the community, which contributes to the development of internalizing symptoms, particularly among girls.

A second major finding was that boys living in previously blockbusted neighborhoods reported less neighborhood cohesion which in turn predicted higher rates of internalizing symptoms. Blockbusted neighborhoods are effectively places of severe white flight and disinvestment (Gotham, 2002). That boys feel less neighborhood cohesion and subsequent elevations in levels of internalizing symptoms here suggests that these places may fail to provide social spaces or engender a sense of community trust (in this case, particularly for boys). Given levels of disinvestment in blockbusted neighborhoods, some such places may also have higher crime rates. Internalizing symptoms among males are worse in high-crime neighborhoods, thus if the two are coincident, it would explain the relation between blockbusting and internalizing symptoms among boys. Further investigation of this potential relationship is warranted.

Our analyses did not find any impacts of redlining on social cohesion or internalizing symptoms. Although the impacts of redlining on inter-generational wealth and other issues remain unresolved, the lack of an association suggests that people physically living in these spaces do not experience significantly worse outcomes than people in other neighborhoods. And while redlining and gentrification were coincident in some cases (Figure 1), blockbusting almost always occurred apart from either of these. In Figure 1, we distinguish extreme blockbusting (>75% of the white population) from high blockbusting (50–75% of the white population) neighborhoods, but they are treated the same in analysis.

Given the significant effects of blockbusting on health outcomes, these findings are important; they illustrate the need for land use policies that address legacy effects of types of housing discrimination beyond redlining. Such understanding is essential for future urban planning approaches that aim to build more equitable cities.

### 4.1. Limitations

Despite these strengths, our findings should be considered in light of a few limitations. First, the study utilized a cross-sectional design, which prevented us from disentangling the temporal relations between neighborhood cohesion and internalizing symptoms. It is possible that youth who experience higher levels of internalizing symptoms may, in turn, experience their neighborhoods as less interconnected. Second, we utilized self-report assessments to capture individuals’ perceptions regarding both neighborhood characteristics and internalizing symptoms, which may have introduced bias related to shared method variance. Subsequent research into these domains may consider using other methods to evaluate neighborhood cohesion, including social network analyses, to more objectively capture these relations. Third, few studies have used an operational GIS-based definition of blockbusting (as in Sadler & Lafreniere, 2017). While this adds important novelty to our findings, it will also be important for future studies to validate further these approaches. Moreover, future research examining the impact of historical discriminatory housing practices on other indicators of health among individuals across the life course is warranted.

### 4.2. Policy Implications and Conclusions

As the fields of public health and urban planning continue their path toward reconnection (Corburn, 2004; Pastor & Morello-Frosch, 2014), we also highlight here several strengths of our work on which future work can build. Our article makes a novel contribution to the literature by examining whether historical neighborhood practices are associated with internalizing symptoms and whether neighborhood cohesion influences the relation between inequitable housing practices and internalizing symptoms in a sample of low-income African American adolescents and young adults.

Specifically, our use of GIS to connect individual participants’ neighborhoods to inequitable housing practices is particularly novel. This approach allowed us to capture objective measures of historical neighborhood characteristics and examine the influence of participants’ perceptions of neighborhood cohesion and mental well-being. Additional strengths of the study include the careful examination of the role of participant sex in study constructs. While other studies suggest that geographic characteristics may impact boys’ and girls’ mental health differentially (Leventhal & Brooks-Gunn, 2003; Popkin et al., 2010), this is the first study to examine neighborhood social cohesion as a potential pathway that may influence associations between discriminatory housidiscriminatory housing practices and internalizing symptom associations as a function of participant sex. Although the examination of individual discriminatory housing practices in relation to youth internalizing symptoms is novel, it is likely that the experience of more than one type of historical discriminatory neighborhood practice may not only shape the physical environment, but also one’s subjective experience of that environment. Future research should leverage person-centered approaches (e.g., latent profile analysis) to identify typologies of historical neighborhood practices and whether these typologies are differentially associated with youth outcomes. Finally, our study examined these processes in a vulnerable sample of adolescents and young adults from disadvantaged neighborhoods. Operationalizing knowledge of the effects of inequitable housing practices can help redevelopment plans to be more intentional in their design and deliberately incorporate aspects that help prevent the onset of or stem the presence of internalizing symptoms and related negative sequelae.

These results have potential policy applications, as they demonstrate the impacts of decades of housing practices on mental health outcomes. These findings highlight the need for considering mental health in determining housing policy (Acevedo-Garcia et al., 2004) and suggest that both historical (blockbusting) and current (gentrification) housing trends impact residents’ well-being. Improving understanding of neighborhood context can help in devising more explicit and effective interventions to ameliorate the negative effects of disinvestment and discrimination. Cities and advocates can leverage our work and other future studies to inform remunerative and regenerative approaches to reinvestment in formerly disinvested communities.

## Figures and Tables

**Table 1.** Characteristics of the analytic sample.

Characteristic	*n* (%)		
Sex			
Male	374 (51.0%)		
Female	359 (49.0%)		
Years of schooling			
Less than 9th grade	98 (13.3%)		
9th grade	188 (25.6%)		
10th grade	174 (23.7%)		
11th grade	143 (19.5%)		
12th grade	98 (13.4%)		
Beyond high school	32 (4.4%)		
General education degree			
Yes	27 (4.5%)		
No	576 (95.5%)		
Employed part- or full-time			
Yes	85 (11.6%)		
No	648 (88.4%)		
Intervention			
Yes	418 (62.1%)		
No	255 (37.9%)		
	*M* (*SD*)	range	*n*
Age	18.76 (1.71)	16–23	733
Neighborhood cohesion	6.76 (2.35)	3–12	719
Blockbusting	0.38 (0.41)	0.00–1.00	706
Gentrification	0.08 (0.23)	0.00–1.00	706
Redlining	0.22 (0.33)	0.00–1.00	706
Depressive symptoms (CES-D)	14.76 (9.89)	0–56	729
Anxiety symptoms (BAI)	6.41 (7.93)	0–63	732

Notes: CES-D—Center for Epidemiological Studies of Depression; BAI—Beck anxiety inventory.

**Table 2.** Unstandardized and standardized beta weights from the final steps of hierarchical models of discriminatory housing practices’ associations with neighborhood cohesion.

Variable	*B*	*SEB*	*β*	*t*	*p*
Intercept	9.85	1.00	—	9.90	<0.001
Sex	−0.22	0.19	−0.05	−1.17	0.243
Age	−0.14	0.05	−0.11	−0.79	0.005
Gentrification	1.65	1.21	0.16	1.37	0.172
Sex × Gentrification	−1.51	0.77	−0.23	−1.96	0.050
Int.	10.66	1.02	—	10.48	<0.001
Sex	−0.84	0.24	−0.18	−3.52	<0.001
Age	−0.14	0.05	−0.10	−2.70	0.007
Blockbusting	−2.11	0.69	−0.37	−3.06	0.002
Sex × Blockbusting	1.33	0.43	0.40	3.11	0.002
Int.	9.71	1.01	—	9.66	<0.001
Sex	−0.19	0.21	−0.04	−0.91	0.365
Age	−0.14	0.05	−0.10	−2.67	0.008
Redlining	0.79	0.88	0.11	0.90	0.368
Sex × Redlining	−0.68	0.54	−0.16	−1.26	0.209

Notes: Sex is coded 1 for male and 2 for female.
